# The Relationship between Red Cell Distribution Width and Residual SYNTAX Scores in ST-Segment Elevation Myocardial Infarction Patients after Percutaneous Coronary Intervention

**DOI:** 10.1155/2021/3281837

**Published:** 2021-12-15

**Authors:** Yang Ling, Wei Wang, Cong Fu, Qun Fan, Jichun Liu, Shengxing Tang

**Affiliations:** ^1^Department of Cardiology, Yijishan Hospital Affiliated to Wannan Medical College, Wuhu 241000, China; ^2^Department of Gastroenterology, Yijishan Hospital Affiliated to Wannan Medical College, Wuhu 241000, China

## Abstract

**Objective:**

Residual SYNTAX score (rSS) values have been suggested to serve as an independent predictor of mortality in ST-segment elevation myocardial infarction (STEMI) patients following percutaneous coronary intervention (PCI). Prior work has also indicated that red cell distribution width (RDW) can predict the incidence of major adverse cardiac events (MACEs) in STEMI patients. As such, we sought to explore the relationship between RDW and rSS in STEMI patients that have undergone PCI.

**Methods:**

In total, 456 eligible patients were recruited for this study. Youden's index was used to calculate the optimal RDW cut-off value, after which the relationship between RDW and rSS values was assessed through Spearman's correlation analyses. Independent predictors of high rSS levels were then identified via multivariate logistic regression analysis.

**Results:**

Patients were separated into two groups based upon whether they exhibited high RDW levels (>13.9, Group 1) or low RDW levels (<13.9, Group 2). The average rSS value of patients in Group 2 was found to be significantly decreased compared to patients in Group 1 (*P* < 0.001). RDW values were found to be positively correlated with rSS (*r* = 0.604, *P* < 0.001), and multivariate logistic regression analysis determined that high RDW levels were independently predictive of higher rSS (OR = 27.1 [14.8-51.7]; *P* < 0.001). Additionally, a nomogram incorporating RDW exhibited good calibration, discriminative capacity, and clinical utility.

**Conclusions:**

In summary, RDW is strongly correlated with rSS in STEMI patients following PCI, with high RDW levels serving as an independent predictor of high rSS in this patient population.

## 1. Introduction

ST-segment elevation myocardial infarction (STEMI), also known as transmural myocardial ischemia, is still the most prominent cause of global morbidity and mortality [1–3]. Percutaneous coronary intervention- (PCI-) based revascularization of the occluded vessel in STEMI patients can dramatically alleviate their symptoms, reduce infarct size, and improve infarct-free survival [4]. However, over 40% of STEMI patients undergoing PCI exhibit multivessel coronary artery disease (MVD) [5, 6], and the incidence of MVD among these patients is positively correlated with higher major adverse cardiac event (MACE) rates and all-cause mortality as compared to STEMI patients with single-vessel disease (SVD) [7, 8]. As such, residual coronary stenoses following PCI have the potential to be extremely detrimental in patients with STEMI and MVD.

The ACUITY trial defined the residual SYNTAX score (rSS) as a tool capable of quantifying and stratifying the degree and complexity of residual coronary stenoses following PCI [9]. Among individuals with moderate- and high-risk acute coronary syndrome (ACS), an rSS > 8 is closely linked to poorer patient outcomes including higher rates of 1-year mortality and MACEs [9]. Additionally, rSS has been shown to independently predict MACE incidence and all-cause mortality in STEMI patients [5, 10].

Red cell distribution width (RDW), defined as the variation in the size of circulating erythrocytes, has been proposed to be a valuable indirect biomarker of inflammation and oxidative stress [11, 12]. Both inflammation and oxidative stress can play an important role in the initiation and progression of unstable plaques, leading to their rupture and consequent thrombus formation [13, 14]. Several studies have further highlighted a significant relationship between higher RDW levels and an elevated risk of death among STEMI patients following PCI [15, 16].

To the best of our knowledge, the relationship between RDW and rSS remains to be investigated. As such, the present study was conceptualized with the following aims: (1) to determine whether RDW is independently associated with rSS in STEMI patients following PCI and (2) to construct a nomogram model incorporating RDW as a tool for predicting high rSS levels in these STEMI patients.

## 2. Materials and Methods

### 2.1. Study Population

**Figure 1:**
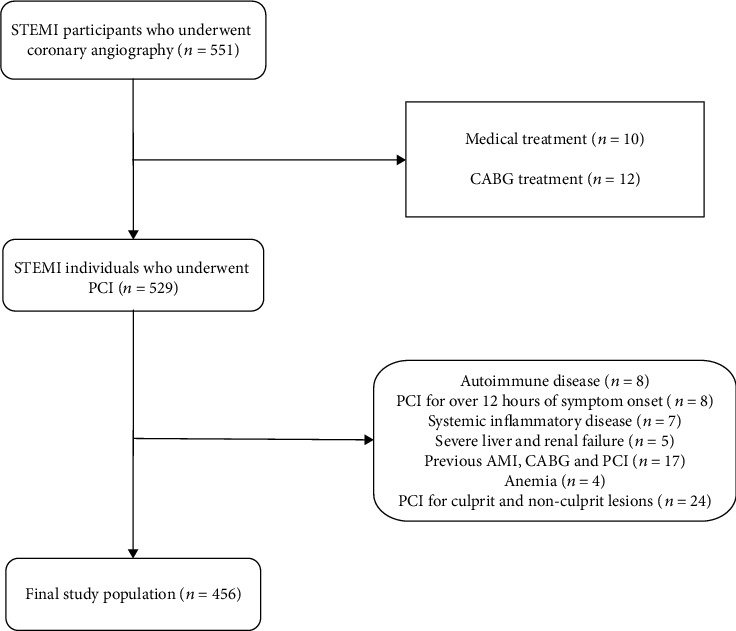
Flow diagram indicating enrollment and exclusions.

We enrolled consecutive individuals who were admitted to our institution with a diagnosis of STEMI between September 2016 and December 2020. The criteria for diagnosing STEMI were based upon the standard defined by the American College of Cardiology, including characteristic myocardial ischemia symptoms accompanied by electrocardiographic ST-segment elevation or detected markers of myocardial necrosis [17]. In total, 551 STEMI patients were initially recruited for this study. All patients that underwent PCI more than 12 h after symptom onset, coronary artery bypass grafting surgery (CABG), or other medical treatments were excluded from this study, as were patients with severe liver or renal disease, active infections, systemic inflammatory disease, autoimmunity, hematological disorders, malignancies, and those with history of hyperlipidemia, chronic obstructive pulmonary disease, anemia, PCI, AMI, CABG, or blood transfusions within 6 months. Following coronary angiography, patients undergoing PCI for both culprit and nonculprit lesions were additionally excluded, yielding a final study population of 456 patients (Figure 1). Given the retrospective nature of the study, the need for informed consent from patients was waived. The Institutional Review Board of Yijishan Hospital Affiliated of Wannan Medical College approved this study.

### 2.2. Patient Characteristics

Demographic and clinical parameters at admission were extracted from the hospital electronic database. All blood samples were collected before patients underwent coronary angiography. Hematologic indices, including hemoglobin, RDW, platelets (PLT), white blood cells (WBC), neutrophils (NEUT), lymphocytes (LMO), monocytes (MO), and eosinophils (EO), were measured with an automated hematological analyzer, while a chemistry analyzer was used to assess biochemical parameters including glucose, total cholesterol (TC), triglycerides (TG), high-density lipoprotein cholesterol (HDL-c), low-density lipoprotein cholesterol (LDL-c), and creatinine. In addition, transthoracic echocardiography was completed immediately before PCI to evaluate left ventricular ejection fraction (LVEF) during hospitalization.

### 2.3. Coronary Angiographic Analysis

Coronary angiography was performed for included patients using the standard Judkins technique via the femoral or radial approach within 90 minutes of admission. The extent and complexity of coronary artery disease was assessed by two experienced interventional cardiologists blinded to patient clinical data [6]. The SYNTAX score (SS), which is a quantitative tool employed to assess the degree of atherosclerosis, was calculated using the online SYNTAX score calculator (http://www.syntaxscore.com, version 2.1). The rSS was utilized to quantitatively assess the extent and severity of residual stenosis by recalculating the SS following the completion of PCI for the culprit lesion. These SS and rSS values were obtained based upon lesions, which were defined as a ≥50% luminal narrowing in a vessel ≥ 1.5 mm [5]. In addition, MVD was defined as at least one lesion with ≥50% luminal stenosis in a major noninfarct-related artery or its branch [8].

### 2.4. Statistical Analysis

Quantitative variables are means ± standard deviation (SD) or median (interquartile range), whereas categorical variables are described as counts and percentages. The Kolmogorov-Smirnov method was used to establish the normality of continuous variables, with normally and nonnormally distributed data being compared using Student's *t*-tests and Mann–Whitney *U* tests, respectively. Chi-squared tests or Fisher's exact test were used to compare categorical variables. Relationships between variables were assessed via Spearman's correlation analyses. Optimal RDW cut-off values associated with higher rSS values were identified via a receiver operating characteristic (ROC) curve analysis and by calculating Youden's index. Univariate and multivariate logistic regression analyses were conducted to identify factors that were independently related to high rSS [18].

A nomogram was developed to predict the odds of high rSS by incorporating all independent predictors identified through the above multivariate analysis. The calibration, discrimination, and clinical utility of the resultant model were then, respectively, assessed with calibration curves, ROC curves, and corresponding area under the curve (AUC) values and decision curve analysis (DCA) approaches. Internal validation of the model was performed via a 1000-resample bootstrap approach, together with the calculation of a corrected Concordance index (C-index). SPSS v. 23 (IBM, USA) and R (v 4.0.2) were used for all statistical testing.

## 3. Results

In total, 456 STEMI patients that had undergone PCI were included in our retrospective analysis. These patients were stratified into two groups based upon the selected RDW cut-off value, including patients with high RDW levels (>13.9, Group 1) and low RDW levels (<13.9, Group 2) (Supplemental file (available here)). Baseline clinical, demographic, and laboratory characteristics of the study population are summarized in Table 1. Group 1 patients were significantly older than Group 2 patients (*P* = 0.002). Additionally, the LVEF of patients in Group 1 was significantly lower than that of patients in Group 2 (48.4 ± 7.1 vs. 51.8 ± 6.6, *P* = 0.032). There were no significant differences in gender, smoking, hypertension, diabetes mellitus, hemoglobin, LMO, MO, EO, glucose, TC, TG, HDL-c, LDL-c, apoB, apoA1, Lpa, or uric acid levels between these groups. With respect to other laboratory variables, WBC (11.5 vs. 10.7, *P* = 0.001), NEUT (9.3 vs. 8.5, *P* = 0.008), BUN (5.8 vs. 5, *P* = 0.005), Cr (77.7 vs. 73.7, *P* = 0.028), and dDimer (0.6 vs. 0.3, *P* < 0.001) were higher in Group 1, whereas PLT (166.0 vs. 180.5, *P* = 0.004) and ALB (36.0 ± 3.9 vs. 36.9 ± 3.9, *P* = 0.021) were lower in Group 1.

**Figure 2:**
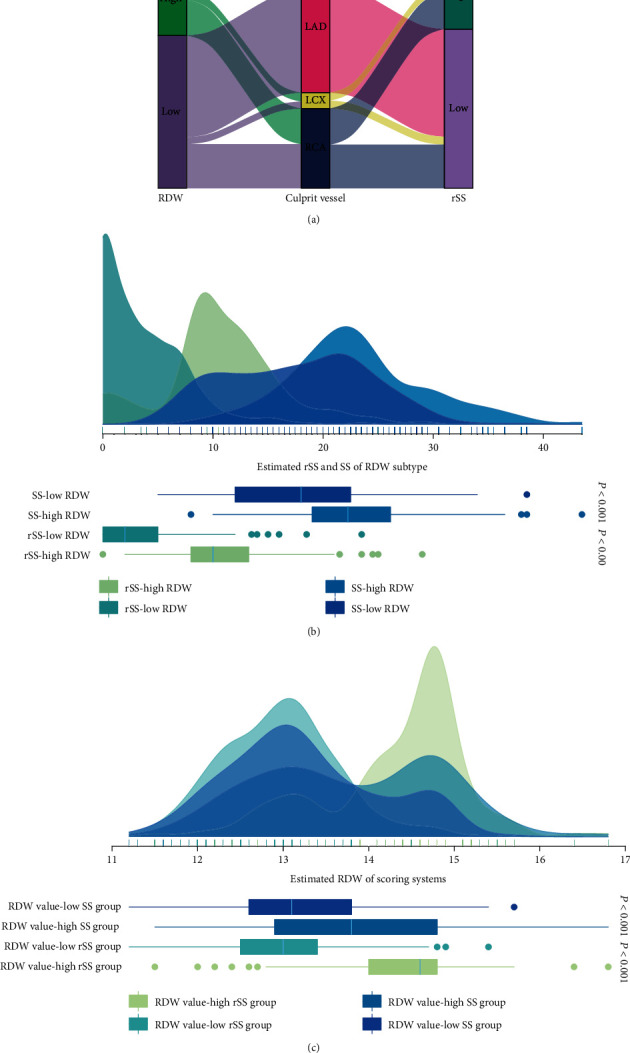
Correlation between the RDW and scoring systems. (a) Alluvial diagram visualizing the connection between RDW, culprit vessel, and rSS. (b) Distribution of rSS and SS in low- and high-RDW subgroups. (c) Distribution of RDW values in rSS and SS subgroups. RDW: red cell distribution width; rSS: residual SYNTAX score; SS: SYNTAX score.

The relevant culprit vessel distributions varied significantly between these groups (*P* < 0.001), with LAD being more common as the culprit vessel in Group 2 relative to Group 1 (Table 1 and Figure 2(a)). The SS and rSS values of patients in Group 2 were also lower relative to those in Group 1 (Figures 2(a) and 2(b)). With respect to the extent of residual coronary stenoses, the percentage of patients with low rSS values was higher in Group 2 compared to Group 1 in all culprit vessel subgroups (Table 2).

**Figure 3:**
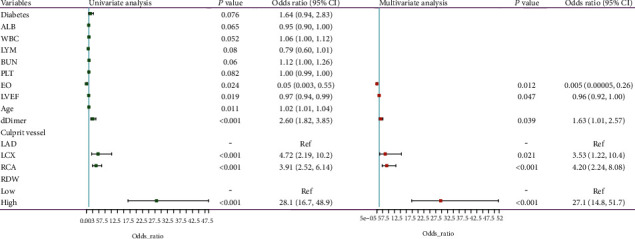
Univariate and multivariate logistic regression analyses of independent predictors of high rSS. rSS: residual SYNTAX score.

**Figure 4:**
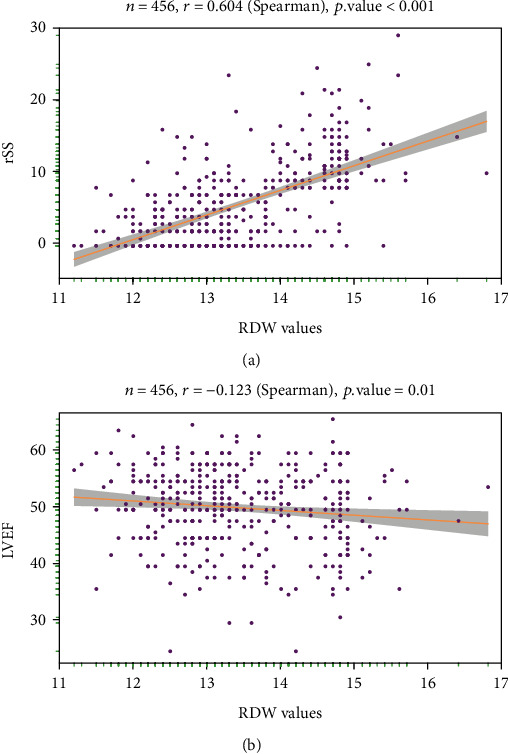
Correlation between RDW and rSS (a) and LVEF (b). RDW: red cell distribution width; rSS: residual SYNTAX score; LVEF: left ventricular ejection fraction.

Logistic regression analyses were conducted to identify independent predictors of high rSS levels (Figure 3), The EO (OR = 0.005 [0.00005-0.26]; *P* = 0.012), LVEF (OR = 0.96 [0.92-1.00]; *P* = 0.047), dDimer (OR = 1.63 [1.01-2.57]; *P* = 0.039), culprit vessel (RCA vs. LAD; OR = 4.20 [2.24-8.08]; *P* < 0.001), and high RDW levels (OR = 27.1 [14.8-51.7]; *P* < 0.001) were all found to be independent predictors of high rSS. Correlation analyses further suggested that RDW values were positively associated with rSS (*r* = 0.604, *P* < 0.001), whereas they were negatively associated with LVEF (*r* = −0.123, *P* = 0.01) (Figures 4(a) and 4(b)).

**Figure 5:**
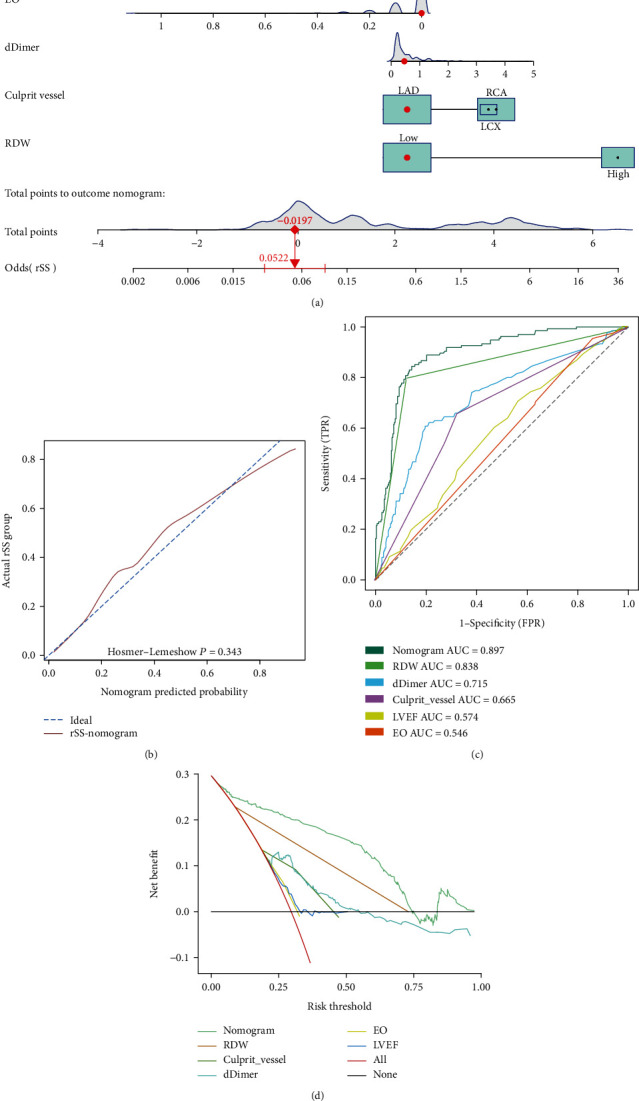
A nomogram incorporating RDW was constructed. (a) Nomogram predicting high rSS of STEMI patients following PCI based on independent predictors. (b) Calibration curve for predicting high rSS in STEMI patients after PCI. (c) ROC curve analysis and comparison of the AUCs for the nomogram, RDW, dDimer, culprit vessel, LVEF, and EO in patients with STEMI. (d) DCA for the prediction model with respect to the use of nomogram, dDimer, culprit vessel, LVEF, and EO. RDW: red cell distribution width; rSS: residual SYNTAX score; STEMI: ST-segment elevation myocardial infarction; PCI: percutaneous coronary intervention; ROC: receiver operating characteristic; AUC: area under the curve; LVEF: left ventricular ejection fraction; EO: eosinophils; DCA: decision curve analysis.

Lastly, we constructed a nomogram incorporating the independent predictors identified through our multivariate logistic regression analysis (Figure 5(a)). This nomogram was evaluated with a calibration curve and the Hosmer-Lemeshow test, which confirmed good model calibration as evidenced by the consistency between nomogram-predicted probabilities of a high rSS and actual observations (Figure 5(b)). ROC curves and a DCA further confirmed that this nomogram was associated with superior discriminatory ability (AUC = 0.897) and more net clinical benefit as compared to these independent factors when assessed in isolation (Figures 5(c) and 5(d)). The internal validation-corrected C-index value was 0.891. Together, these findings confirmed the satisfactory performance of this nomogram as a tool for predicting the risk of high rSS.

## 4. Discussion

In this report, we detected a positive relationship between RDW and rSS among STEMI patients following PCI, and we further determined high RDW levels to be an independent predictor of high rSS. Based on these findings, we then constructed a nomogram that incorporated RDW and exhibited good performance when predicting high rSS among a STEMI patient population.

Both oxidative stress and inflammation have been linked to the onset, progression, and ultimate prognosis of coronary atherosclerosis by promoting plaque instability, plaque rupture, and consequent thrombus formation [13, 14]. MVD patients account for over 40% of all STEMI patients following PCI and are at an elevated risk of cardiac death and MACEs [6–8]. The optimal treatment for residual stenoses after infarct-related artery reperfusion in STEMI patients remains a topic of debate. As an indicator of residual disease burden following PCI, rSS was found to independently predict the risk of cardiac death and MACEs in the ACUITY trial [9]. Sonya et al. reported the rates of cardiac death and myocardial infarction (MI) for MVD and STEMI patients with an rSS of 0, 1-8, and >8 of 5%, 15%, and 26%, respectively, during follow-up, with an association between an rSS > 8 and the higher rates of cardiac mortality and MI [10]. In a retrospective cohort study including STEMI and MVD patients following primary PCI, rSS was found to be not only positively correlated with all-cause mortality and MACE but also an independent predictor of these outcomes during follow-up [5].

RDW, which is also referred to as anisocytosis, serves as a measure of the variability in erythrocyte size distributions and can be readily analyzed with an automatic hematological analyzer [19]. RDW is an indirect marker of inflammation and oxidative stress [11, 20], with most pathological conditions having been linked to an increase in RDW. Zalawadiya et al. performed a cross-sectional study identifying an independent association between RDW and the risk of peripheral arterial disease [21], while another cohort study of 27,124 participants followed for an average of 13.6 years detected an association between RDW and incidence of atrial fibrillation [22]. Several studies have also reported an independent association between RDW and adverse cardiovascular disease-related events including heart failure, chronic coronary artery disease (CAD), STEMI, and non-STEMI [15, 23–27]. A strong independent correlation between the level of RDW and the noninfarct-related artery in AMI patients has also been proposed [28]. Akboga et al. demonstrated that RDW was able to independently predict the occlusion of the infarct-related artery in STEMI patients [20], and a higher RDW was found to be independently associated with a higher risk of in-hospital mortality in STEMI patients following PCI [29]. Nabais et al. similarly presented a link between elevated RDW levels and mortality or MI in ACS [30]. Furthermore, RDW has been reported to be independently correlated with the presence and extent of CAD in patients with AMI and stable angina pectoris as evaluated by the SS [27, 31]. In this report, we found that higher RDW levels were more common among STEMI patients with a high rSS and that increased RDW levels were independently predictive of high rSS. We additionally constructed a nomogram model incorporating RDW and other parameters to predict high rSS and found this nomogram to exhibit satisfactory discrimination, calibration, and clinical utility.

We have demonstrated a positive correlation between RDW and the degree and complexity of residual coronary stenosis in STEMI patients following PCI. We also found that high RDW values on admission are an independent predictor of a high rSS. The underlying mechanisms governing the relationship between RDW and rSS are as follows. First, inflammation plays a vital role in the atherosclerotic process [13]. A prior study has suggested that there is a positive association between RDW and both high-sensitivity C-reactive protein levels and erythrocyte sedimentation rate, both of which are inflammatory biomarkers [32]. The association between RDW and interleukin-6 or soluble tumor necrosis factor receptor I or II have been reported in several studies [12, 33]. These results have proven the association between generalized inflammation and increased RDW values. In addition, inflammation can also induce aberrant iron metabolism and decreases in erythropoietin production, in turn impairing erythrocyte maturation and ultimately increasing RDW levels [19, 34]. Moreover, oxidative stress further induces the transformation of LDL cholesterol into oxidized-LDL, which is responsible for boosting the inflammatory response and the progression of atherosclerosis [35]. Additionally, oxidative stress has also been shown to directly regulate the lifespan of erythrocytes, promoting both the production and the release of various cellular structures into circulation, thereby leading to increases in RDW values [36, 37]. Semba et al. have proposed that the serum selenium level, as an indicator of the degree of antioxidant activity, is an independent predictor of RDW and may exert a crucial effect on the correlation between high RDW levels and a poor prognosis [38]. The association between RDW and rSS can thus be linked to processes underlying both of these metrics including oxidative stress and inflammation.

This study is subject to a few limitations that warrant consideration. First, we examined the association between the rSS and RDW; however, it is difficult to make causal inferences due to the nature of the cross-sectional design. Second, this was a retrospective single-center analysis without the potential to avoid selection bias. Several confounding factors might have affected the results even after the adjusted analysis. Additionally, no biomarkers directly linked to inflammation or oxidative stress were analyzed herein. External validation will be essential to confirm these results.

## 5. Conclusion

In conclusion, we observed a positive correlation between RDW and rSS in STEMI patients following PCI, with high RDW levels being an independent predictor of high rSS, thus suggesting that RDW may be a valuable and easy-to-assess biomarker that can guide the risk stratification of STEMI patients following PCI.

## Figures and Tables

**Table 1:** Baseline clinical, demographic, and laboratory characteristics of the study population.

Characteristics	All patients (*n* = 456)	High RDW (*n* = 148)	Low RDW (*n* = 308)	*P*
RDW	13.3 (12.7-14.3)	14.7 (14.3-14.8)	12.9 (12.4-13.3)	<0.001
Age (years)	63 (53-72)	65.5 (56-74)	62 (52-71)	0.002
Gender (female), *n* (%)	91 (20)	28 (18.9)	63 (20.5)	0.701
Smoking, *n* (%)	253 (55.5)	87 (58.8)	166 (53.9)	0.325
Hypertension, *n* (%)	226 (49.6)	71 (48)	155 (50.3)	0.638
DM, *n* (%)	64 (14)	25 (16.9)	39 (12.7)	0.223
Hb (g/L)	142.0 (129-154)	141.0 (126-152.8)	142.0 (130-155)	0.183
WBC (10^9^/L)	10.9 (9.1-13)	11.5 (9.9-14.1)	10.7 (8.7-12.7)	0.001
NEUT (10^9^/L)	8.8 (6.9-11.1)	9.3 (7.3-12.2)	8.5 (6.7-10.7)	0.008
LYM (10^9^/L)	1.2 (0.8-1.7)	1.1 (0.8-1.6)	1.2 (0.9-1.7)	0.194
MO (10^9^/L)	0.4 (0.3-0.6)	0.4 (0.3-0.6)	0.4 (0.3-0.6)	0.737
EO (10^9^/L)	0 (0-0.1)	0 (0-0.1)	0 (0-0.1)	0.654
PLT (10^9^/L)	173.0 (138-218.8)	166.0 (125-209.8)	180.5 (144.2-225.8)	0.004
Glucose (mmol/L)	5.5 (4.7-6.9)	5.6 (4.7-7.1)	5.5 (4.7-6.8)	0.377
TC (mmol/L)	4.1 (3.5-4.8)	4.1 (3.3-4.8)	4.1 (3.6-4.8)	0.582
TG (mmol/L)	1.3 (0.9-1.9)	1.3 (0.9-1.8)	1.3 (1.0-2.1)	0.26
HDL-c (mmol/L)	1.2 (1.0-1.3)	1.2 (1.0-1.3)	1.2 (1.0-1.3)	0.515
LDL-c (mmol/L)	2.3 (1.9-2.8)	2.4 (1.8-2.9)	2.3 (2.0-2.8)	0.827
apoB (g/L)	0.9 (0.7-1.0)	0.8 (0.7-1.0)	0.9 (0.7-1.0)	0.295
apoA1 (g/L)	1.1 (1.0-1.3)	1.1 (0.9-1.2)	1.1 (1.0-1.3)	0.181
Lpa (mg/L)	217.2 (102.5-384.8)	233 (105.8-429.8)	209.8 (101.7-368.9)	0.252
UA (*μ*mol/L)	351.5 (285.9-427.9)	353.6 (290.8-443.7)	350.6 (282.5-421.5)	0.447
BUN (mmol/L)	5.3 (4.2-6.4)	5.8 (4.6-6.6)	5.0 (4.1-6.3)	0.005
Cr (*μ*mol/L)	74.7 (62.3-90.9)	77.7 (64.9-94.3)	73.7 (61.2-88.9)	0.028
ALB (g/L)	36.6 ± 3.9	36.0 ± 3.9	36.9 ± 3.9	0.021
dDimer (*μ*g/mL)	0.3 (0.19-0.66)	0.6 (0.3-1.1)	0.3 (0.2-0.5)	<0.001
LVEF (%)	50.3 ± 6.8	48.4 ± 7.1	51.8 ± 6.6	0.032
Culprit vessel, *n* (%)				
LAD	263 (57.7)	59 (39.9)	204 (66.2)	<0.001
LCX	32 (7)	18 (12.2)	14 (4.5)	
RCA	161 (35.3)	71 (48.0)	90 (29.2)	

Abbreviations: DM: diabetes mellitus; Hb: hemoglobin; WBC: white blood cells; NEUT: neutrophils; LYM: lymphocytes; MO: monocytes; EO: eosinophils; PLT: platelets; RDW: red blood cell distribution width; TC: total cholesterol; TG: triglycerides; HDL-c: high-density lipoprotein cholesterol; LDL-c: low-density lipoprotein cholesterol; Lpa: lipoprotein a; UA: uric acid; BUN: blood urea nitrogen; Cr: creatinine; ALB: albumin; LVEF: left ventricular ejection fraction; LAD: left anterior descending; LCX: left circumflex; RCA: right coronary artery.

**Table 2:** The extent of residual coronary stenoses based on culprit vessels.

Culprit vessels	All patients (*n* = 456)	High RDW (*n* = 148)	Low RDW (*n* = 308)	*P*
LAD, *n* (%)	263 (100)	59 (22.4)	204 (77.6)	—
rSS	2 (0-6)	9 (6-12)	2 (0-4)	<0.001
rSS group, *n* (%)				<0.001
Low (≤8)	217 (82.5)	22 (10.1)	195 (89.9)	
High (>8)	46 (17.5)	37 (80.4)	9 (19.6)	
LCX, *n* (%)	32 (100)	18 (56.3)	14 (43.7)	—
rSS	8.5 (5-12)	11.5 (9-13.5)	5 (1.5-7)	<0.001
rSS group, *n* (%)				<0.001
Low (≤8)	16 (50)	2 (12.5)	14 (87.5)	
High (>8)	16 (50)	16 (100)	0 (0)	
RCA, *n* (%)	161 (100)	71 (44.1)	90 (55.9)	—
rSS	8 (2-12)	12 (9-14)	5 (0-8)	<0.001
rSS group, *n* (%)				<0.001
Low (≤8)	88 (54.7)	16 (18.2)	72 (81.8)	
High (>8)	73 (45.3)	55 (75.3)	18 (24.7)	

Abbreviations: RDW: red blood cell distribution width; LAD: left anterior descending; LCX: left circumflex; RCA: right coronary artery; rSS: residual SYNTAX score.

## Data Availability

The raw data supporting the conclusions of this article will be made available by the corresponding authors, without undue reservation.

## Supplementary Materials

Supplementary Materials Supplemental Figure 1: Youden's index was used to calculate the optimal RDW cut-off value. Receiver operating characteristic (ROC) curve analysis displays the discriminative ability of the red cell distribution width. IRB: Scientific Research and New Technology of Wannan Medical College Yijishan Hospital Institutional Review Board.
