# Supplementary figures and images for: The Relationship between Red Cell Distribution Width and Residual SYNTAX Scores in ST-Segment Elevation Myocardial Infarction Patients after Percutaneous Coronary Intervention
Source: Dis Markers. 2021 Dec 15;2021:3281837. doi: 10.1155/2021/3281837 (PMC8695033; doi:10.1155/2021/3281837)

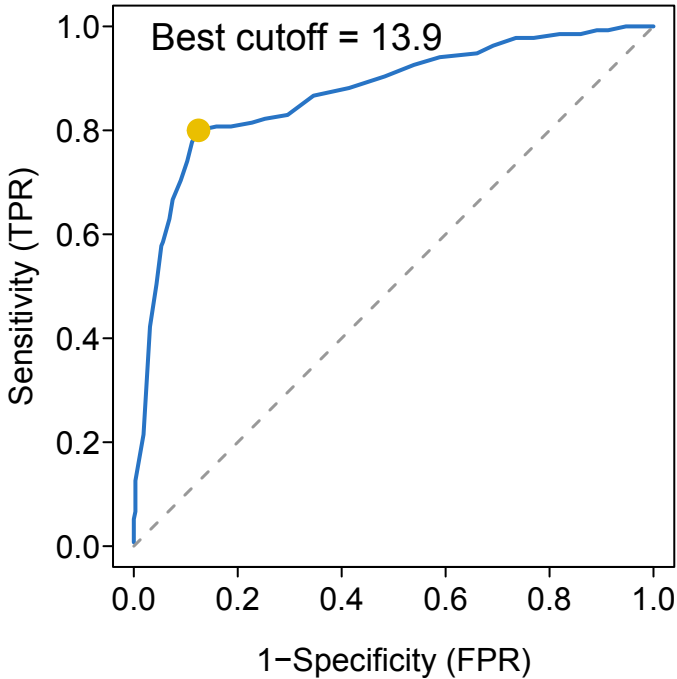

Supplement: Supplementary Materials — Supplemental Figure 1: Youden's index was used to calculate the optimal RDW cut-off value. Receiver operating characteristic (ROC) curve analysis displays the discriminative ability of the red cell distribution width. IRB: Scientific Research and New Technology of Wannan Medical College Yijishan Hospital Institutional Review Board. [file 3281837.f1.pdf]
